# Moving on From the Delphi Study: The Development of a Physical Activity Training Programme Prototype Through Co-produced Qualitative Research

**DOI:** 10.1177/10497323221126535

**Published:** 2022-09-14

**Authors:** Javier Monforte, Chris Davis, Shaesta Saleem, Brett Smith

**Affiliations:** 1 Department of Sport and Exercise Sciences, 3057 Durham University, Durham, UK; 2 Departamento de Educación Física y Deportiva, Universitat de València, València, Spain; 3 We can Move, Active Gloucestershire, Gloucester, UK; 4 Learning Disabilities, Autism and Mental Health, Lancashire County Council, Lancashire, UK

**Keywords:** co-production, dialogical inquiry, data prompted interviews, dyadic interviews, online interviews, social work, physical activity promotion

## Abstract

This research developed from a co-produced project called Moving Social Work. The purpose of this ongoing project is to train social workers in how to promote physical activity for and to disabled people. The first stage of the project consisted of building evidence to design a training programme prototype. As part of this stage, a Delphi study was conducted to ask leading experts about what should be included in the prototype. Questionnaires were sent to participants until consensus was reached. In reflecting on the results, people involved in the study commented that there was more about the experts’ opinions than percentages of agreement. Our co-production partners resolved that the Delphi was insufficient and called for detailed conversations with the experts. In response to this call, follow-up interviews with 10 experts who participated in the final questionnaire round of the Delphi were carried out. The interviews were co-produced, dyadic and data prompted. Dialogical inquiry was used to frame and co-analyse data. The results illuminate the capacity of qualitative research to justify, rectify, complicate, clarify, concretize, expand and question consensus-based evidence. The implications of the results for Moving Social Work are discussed. Beyond the empirical border of the project, wider contributions to literature are presented. As part of these, two key statements are highlighted and warranted: dialogical inquiry supports the practice of co-produced research, and Delphi studies should be followed by a Big Q qualitative study.

## Introduction

In England, four in ten disabled adults feel that they can do as much physical activity (PA) as they want, and eight in ten would like to do more PA (Activity Alliance, 2022). These facts suggest that disabled people face multiple barriers to getting and staying physically active. Frequently reported barriers to PA include high costs, ableist environments, and lack of transportation and equipment (Jaarsma et al., 2019; Mascarinas & Blauwet, 2018). However, another significant blocker is the insufficient flow of PA information, meaning that valuable PA information is not reaching the people who are looking to access it (Jaarsma et al., 2019). Improving the flow of PA information is not easy. But one of the keys is identifying trusted collectives of influencers known as messengers, and then helping these messengers understand the target audience and use different types of delivery methods.

Healthcare professionals (HCPs) have been consistently identified as a key PA messenger collective, and several interventions have been established to support them promoting PA (Brannan et al., 2019; Vishnubala & Pringle, 2021). However, HCPs are not the only workforce that can and should have conversations about PA with disabled people. Under the umbrella of a co-produced project called ‘Get Yourself Active’, a study found that disabled people also view social workers (SWs) as credible and desirable messengers. As the authors reflected:

The identification of SWs was significant as they had not been highlighted as a key messenger group before by disabled people in the physical activity literature. Not only was this the first study in which SWs had been identified by disabled people as key physical activity messengers, but disabled people also often viewed SWs as “better” messengers than health professionals. ((Smith & Wightman, 2019), p. 3428)

Extending the cited study, Smith et al. (forthcoming) established nine evidence-based reasons why SWs should promote PA for disabled people. Two of such reasons are that HCPs do not want to be the only professional group of PA messengers, and that interprofessional collaboration between health and social care professionals is more effective. Despite the existing rationale, SWs remain largely unaware of their potential as PA messengers and have not received any training in order to develop their knowledge, confidence and skills. This means that a good opportunity to support disabled people reach relevant PA information is currently being missed.

To initiate a paradigm shift, a project funded by Sport England and the National Institute for Health Research called ‘Moving Social Work’ (MSW) was launched. Set in the UK, the purpose of this ongoing project is to provide structured training and education for the SWs of today and tomorrow on how to successfully promote PA for disabled people. MSW is an ‘Equitable and Experientially-informed’ co-production project (Smith et al., 2022). This means that equitable partnerships between different people with relevant lived experience shape the research from beginning to end. Conceived together with The Moving Social Work Co-production Collective, the first stage of MSW consisted of building evidence to inform the design of a training programme prototype. To do so, two studies were designed.

The first study was a scoping review. With this, we learned valuable lessons about how HCPs have been and are being trained in PA promotion, specifically what contents they are taught and how (Netherway et al., 2021). Although this knowledge was useful, we could not merely transport what has been done in the realm of health care to social care. General practitioners, for example, and SWs, have different professional standards and interests. Partners from the co-production group, including but not limited to disabled people and SWs, highlighted the need of taking these differences into account, and called for training resources that suit the skills of SWs, as well as their professional ethos and culture.

Considering the foregoing, the second study aimed to determine which culturally appropriate contents and teaching methods should be used in the training programme prototype. Additionally, it aimed to identify what are the potential barriers that could jeopardise the intended success of the programme in action. To conduct such a study, we used a Delphi method. This method has been recommended for curriculum design in higher education since the 70’s (Reeves & Jauch, 1978). Moreover, recent studies used it to develop training programmes in PA promotion. For instance, Wattanapisit et al. (2019) used it to identify and prioritise key elements for PA counselling in medical education, arguing that ‘the characteristics of the Delphi study, using a series of questionnaires, helped to achieve the consensus of expert opinion and avoid problems arising from a few powerful participants and group pressures’ (p. 1).

**Figure 1.**
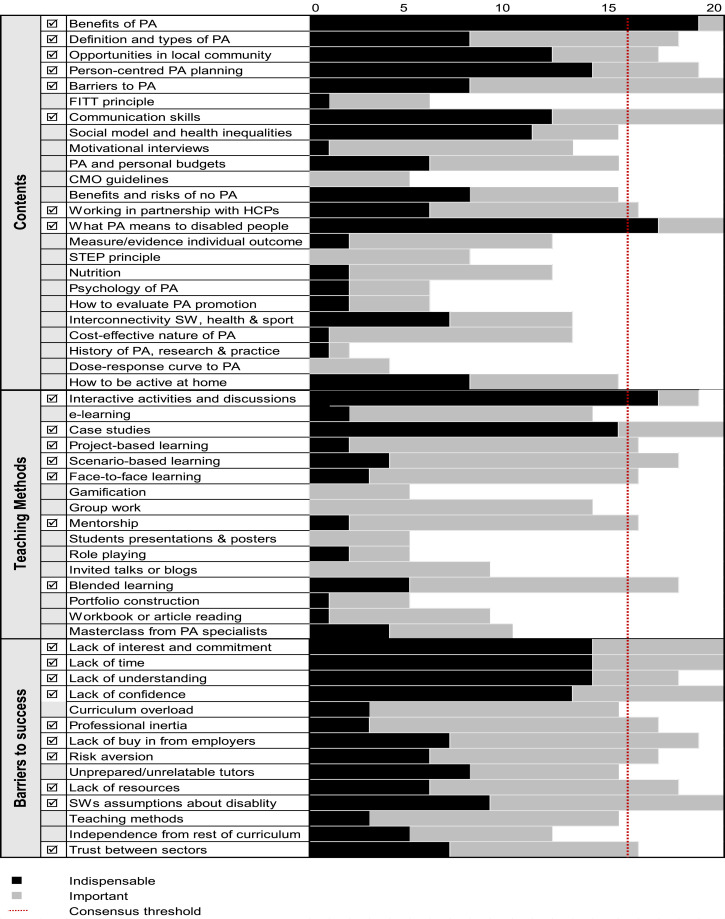
The Delphi study results. Modified from Monforte et al. (2022). To interpret the figure, please consider the following. An item reaches consensus when it is deemed important or indispensable by at least 80% of the experts, namely 16 experts or more. The number of experts that deemed an item indispensable are represented in black. The number of experts who regarded an item as important is indicated in grey. To exemplify, the item “Benefits of PA” reaches consensus because a 100% of experts considered this item either indispensable (95% of them) or important (5%). That is why there is a ☑ next to it. In contrast, the item “Nutrition” did not reached consensus because just 12 experts considered it indispensable (10%) or important (50%).

Participants selected for our Delphi study included experts in physical activity and health, social work and disability, with some experts being experts in two or all these domains, and having experiential knowledge (e.g. having a long-term impairment). Sixty experts were initially recruited, and twenty filled in the third and last questionnaire round. The results of the Delphi study are published in Monforte et al. (2022) and summarised in the Figure 1. This figure will have an important role in this article for reasons revealed later.

As can be observed in the Figure, 8 contents, 7 teaching methods and 10 potential barriers crossed the established consensus threshold in the Delphi study. According to the logics of the Delphi method, the training programme prototype would be composed of the mentioned items. In parallel, including the items that did not reach consensus (i.e. those that were not rated as important or indispensable by at least 80% of the experts in the last round) would not be a priority or would not be recommended.

The evidence from this Delphi study was regarded by people involved in the project as incredibly useful insofar as it offered more specific elements of PA promotion training than the available literature. Simultaneously, however, the study provoked a slight but significant dissatisfaction among researchers, experts and co-production partners.

First, researchers involved in the project reflected that the Delphi atomized those who participated in it, and that access to the rationale underpinning their preferences was limited. Second, although some experts said to us that they ‘enjoyed the process’ and ‘learned from it’, others were critical with the Delphi. For example, one expert commented: ‘I didn’t like it. I’m much happier talking about the issues… I can see why you would do it because presumably that [i.e. the Delphi Method] does fit some people, but it doesn’t fit me’. Similarly, another participant said: ‘I found the [Delphi] process a bit limited in terms of what you could say (…) It didn’t feel like I was getting into the depth of why we were making our points’. Finally, some members of the advisory board and the co-production collective of the project said that they were interested in knowing more about the experts’ ideas and why they left some key content outside consensus. Overall, everybody was on the same page: conducting a follow-up study on expert opinions was pertinent.

In a focus meeting with the Moving Social Work Co-production Collective, we considered the practical consequences that this new research could have for the whole project. Some suggested that the study would help us constructing a more detailed and coherent training programme prototype. Others highlighted the usefulness of gathering specific recommendations on how to apply the identified teaching contents and methods to real practice. Admittedly, however, much of the interest about doing this research was curiosity driven. We all craved to hear the voices of the experts and know more about the background and meaning of their opinions. The question we asked ourselves was: What would we find out if we talk to the experts? However imprecise, this became our research question.

## Methods and Methodology

### Philosophical Stance

The design of this study is underpinned by ontological relativism (i.e. reality is multiple and mind-dependent) and epistemological constructionism (i.e. knowledge about reality is constructed and subjective). More concretely, the study is inspired by dialogism, which assumes that individuals are relational beings who construct knowledge through an open-ended dialogue with other people (Frank, 2005). As Wells et al. (2021) suggested, a dialogical approach can be particularly suitable for research teams that wish to ‘privilege the voices of co-researchers from diverse social, political, and epistemic positions’ or, put differently, to ‘democratize expertise by recognizing various kinds of knowledge’ (p. 499). As such, dialogism serves as a coherent philosophical base for co-production processes, insofar as it opens a dialogical space that allows academics and non-academics establishing equitable partnerships and working together in the production of knowledge.

### The Co-Researchers and Their Critical Friends

As highlighted, members of the MSW co-production group expressed their willingness to know more about the Delphi experts’ views and called for qualitative interviews to generate further knowledge. Accordingly, they were asked if, and how, they would like to be involved in the interview study.

Javier, the lead author of the Delphi study, prepared an easy-read document explaining what becoming a co-researcher in this study would involve. That document was largely inspired by (Smith et al., 2022) as well as the published and unpublished work of Liddiard et al. (2019). Additionally, Javier organised a drop-in session with people in the co-production group to clarify any doubt amongst those interested. Two members of the group, *SecondAuthor* and *ThirdAuthor*, expressed their willingness to participate. *SecondAuthor* is a carer and a sport, health, and leisure professional who works in a local community to help disabled people get active. Meanwhile, *ThirdAuthor* is a qualified social worker whose work has focused on supporting disabled people. The remaining co-production members (including disabled people, activists, social work lecturers, students and professionals, and physical activity champions) agreed to be ‘critical friends’ of the interview study, that is, to offer their feedback and challenge the work by the core research team comprised by the first three authors. (Smith et al., 2009), the lead investigator of the MSW project and an expert on qualitative interviewing, dialogism and co-production (see blinded-for-peer-review), joined the study as a critical friend too. Hence, the rigor of this study is enhanced by two kinds of critical friends: key people with relevant lived experience or experiential knowledge, and a prominent scholar with relevant academic knowledge.

### Deciding on the Style of Interviewing

In a series of videocalls, the three leading co-researchers dialogued about how to collect interview data, and what type or combination of types of interviews could be used. Resulting from this dialogue, different decisions were made. First, the interviews would be carried out via Zoom. This was an easy resolution insofar as both the experts and the co-researchers lived in different geographical locations across the UK. Javier reviewed recent literature on the challenges, opportunities and recommendations in Zoom interviewing (e.g. Archibald et al., 2019; Fouda, 2020; Oliffe et al., 2021). Then, he discussed key points with Chris and Shaesta who, being Zoom users themselves, clearly understood the concessions involved in having conversations through videoconference.

Second, it was agreed data-prompted interviews would be used. This method refers to the use of data gathered prior to the interview as a way of stimulating and facilitating discussion during the interview (Kwasnicka et al., 2015). Namely, it was decided to use as a prompt the Delphi results and, more concretely, the Figure 1 displayed earlier in this paper. This would be displayed during the interviews using the ‘share screen’ function of Zoom. Finally, the three co-researchers decided to conduct dyadic interviews, which involve two, as opposed to one single interviewee in each interview. It has been argued that this kind of interview combine some of the advantages of the focus group interview (e.g. the opportunity for participants to support and prompt each other) while reducing some of its drawbacks (e.g. the limited access offered by larger groups to detailed responses from each participant) (Caldwell, 2014; Morgan et al., 2013). In our context, dyadic interviews were chosen to originate dialogue between experts falling under different areas of expertise (e.g. physical activity/social work). In a Delphi study, experts are free of direct interaction with other experts, and thus their views are to be evaluated on their merit only (Hirschhorn, 2019). In dyadic interviews, the logic is inversed: the views of an expert are created from interaction with another expert and evaluated in relation the other expert’s views.

Following the above decisions, Javier, Chris and Shaesta co-designed an interview guide. Figure 1 was its central component. As visual methods scholars have discussed, the idea is that using a visual material (such as a figure) as a prompt ‘may be more linguistically flexible than an interview schedule’, in that discussion of the figure ‘can pave the way for wider dialogue’ (Leonard & McKnight, 2015, p. 632). The figure would be presented to the experts, and the principal task of the interviewers would be facilitating discussion around it. Shaesta showed concern about the complexities of this task and the realisation that every interview would be different from the rest, and largely unpredictable. She asked: ‘What if we do not know what to ask? Wouldn’t it be better to have some prepared questions?’ Following further conversation, ‘pocket questions’ were designed to support the interviewing process (Smith & Sparkes, 2016).

### Participants and Recruitment

The participants of this study are key experts that took part in all the rounds of our previous Delphi study. At the end of the Delphi, we asked them to indicate if they would be willing to participate in a follow up research. From a group of 20 participants, six voiced their readiness to participate. As Malterud et al. (2016) sustained, the more relevant information a sample has, the fewer participants are needed. However, despite that the six highly qualified and influential experts alone could (arguably) provide very rich information, we hoped to get a more varied range of dyads. Hence, we persevered until four more participants accepted. The final sample of 10 experts equals the 50% of the sample that responded to all the questionnaire rounds of the Delphi study.

Our first attempt was to assign the dyads purposively through using Doodle, a web-based scheduling tool, useful to set up meetings with team members and participants. To start with, we created two Doodle surveys and sent them to two pairs of experts. None of the schedules matched. At this point, we realised about the actual complexity of managing five schedules (three co-interviewers and two busy interview participants per interview). We recovered from this recruiting failure (Clark & Sousa, 2020) by setting another strategy, which consisted of asking all the participants to fill a single doodle. One expert emailed us to express that she changed her mind; she preferred to participate in the next participatory stage of the project, instead of the interview study. Thus, another effort was made to recruit one more participant. Eventually, the dyads were formed based on availability. Relevant information about the participants (names are pseudonyms) is shared in Table 1. The participants gave their written consent to voluntarily partaking in this study and were offered a £20 thank you voucher conditional to the interview completion. The study has the ethical approval of Durham University (SPORT-2020-02- 18T17_18_37-dmgf98).

**Table 1.** The Participants.

Dyad	Name	Pronouns	Expertise	Years of experience
1	Sean	He/Him	Sean is the funder of a leisure company that works with social care coordinators and workers to help disabled people get active using direct payments.	>20
	Bob	He/Him	Bob works in a local council to promote inclusive sport and PA for disabled people. Bob was born with a physical impairment, which means that he has more than 30 years of lived experience.	>20
2	Sue	She/Her	Sue is a world-leading professor of education, and the principal carer of a person with intellectual disability.	>20
	Tim	He/Him	Tim is the president of an international disabled sport association that helps thousands of disabled people enjoy being active.	<20
3	Eva	She/Her	Eva is a PA champion. She works for the UK government and she is the CEO of a health promotion initiative.	>20
	Ceri	She/Her	Ceri is the head of disability in a public sector organisation. She leads several projects to increase the PA levels of disabled people.	<10
4	Rafa	He/Him	Rafa is the director of a nation-wide charity that promotes inclusive PA through community engagement programmes.	>20
	Navi	She/Her	Navi is a social worker involved in an adult social care team. She is interested in how can social workers to promote PA among disabled people and other population. She is also a CPD promoter.	<10
5	Ben	He/Him	Ben is a regional locality manager of a charity for adult social care in the UK.	>20
	Fiona	She/Her	Fiona is a PA champion. She is a community manager in a leisure charity where she focuses on promoting recreational and health-enhancing PA.	>20

### Using Multiple Interviewers

The possibility of using multiple interviewers in qualitative research is not new. Bechofer, Elliott and McCrone (Bechofer et al., 1984) suggested that involving more than one interviewer can provide a greater sense of a casual conversation rather than a formal interview, but also facilitate issues such as observing reactions, changing the subject, and employing diverse interviewing tactics throughout. Although challenging, group interviewing was suitable for this co-produced research, which intended to include the three main co-researchers in every research stage. Initially, the three co-researchers were available to conduct the interviews altogether. However, Shaesta could not participate in the first interview given the incompatibility of her agenda and the experts’. She participated in the next two interviews, but further job obligations first and unexpected personal problems later made impossible for her to keep involved in the remaining data collection. Largely, then, the work was conducted by two interviewers. Recent literature has signalled the potential affordances of using two interviewers and offered diverse recommendations (Monforte & Úbeda-Colomer, 2021; Velardo & Elliott, 2021). For example, Monforte and Úbeda-Colomer suggested that it is important for the interviewers to keep a constant dialogue between interviews in order to attune with one another or – as often phrased in dialogical inquiry – to ‘fusion horizons of understanding’ (Frank, 2011). Consistent with this suggestion, Javier and Chris interchanged emails on a regular basis and met via Zoom before and after each interview to exchange ideas and broad each other’s perceptions about the study.

The constant exchange between the two interviewers not only helped in developing the data collection process but also impacted on upcoming tasks such as the analysis. That became obvious when, one week after the last interview, we met to discuss potential issues of power and authority that could arise during the analytical process. This discussion was guided by some questions inspired by criteria for judging the quality of co-produced research (Smith et al., 2022), such as: Did non-academic researchers feel that their contributions were genuinely engaged with and made a difference to the decisions that were made? To what extent did they believe their personal skills and insights contributed to the research and were valued? Was power shared between academic and non-academic researchers? In engaging with these questions, Chris expressed:

I had my own assumptions and bias, you know, there is an academic in the room… there is an immediate feeling of, historically, throughout my own education, that an academic is always somebody that knows more than me. But when we talk about co-production, as we conducted each interview, my perception of the power has changed. It has levelled out to the point now where I think, you know, we got a really good levelled balanced relationship in terms of academic-non-academic, or just two people.

Certainly, that power differentials are not perceived does not mean that they do not exist – concepts such as symbolic violence (Bourdieu, 1998) help us understand that. Simply put, symbolic violence is an imperceptible but effective form of violence that is exercised upon a person with his or her complicity. Its effectiveness lies in its misrecognition: people subjected to symbolic violence may be subjected to unequal power relationships but not recognise them as such. Put in context, this means that perceptions of power balance from Chris should not be considered an evidence of actual power balance, since inequities in power may remain invisible for him. Having said that, co-production is about trusting, not invalidating, people’s perceptions. Disregarding or invalidating Chris' image of an equal partnership would also be a form of symbolic violence. So, what is to be done? From our perspective (Smith et al., 2022), the soundest way out is to maintain honest conversations regarding how co-researchers ‘are working together, how they respond to conflicting views, and how their assumptions, power, and lived experiences influence the conversations’. Consistent with this perspective, Javier and Chris agreed to keep exchanging challenging questions about their positionality during the whole analytical process.

### Dialogical Analysis

Wells et al.’s (2021) proposed seven dialogical inquiry steps to conduct a ‘receptive, open-ended process of meaning-making’ among a team of three persons or more. However, Shaesta could not join the analysis stage either as her personal issues persisted. This left us with two analysts and therefore with the need of finding or crafting another analytical process. Eventually, Javier found a model detailed in Hermans (2006) but originally formulated by Marková (1987) and Linell and Marková (1993). Although the model serves the purpose of researching the dialogical self between different self-positions (as opposed to different people), the three steps of the model were applicable to our dialogical relationship. Adapted to our context, the steps read as follows:

Step 1: A to B. One co-researcher directs a statement to the other co-researcher. For example, co-researcher A may state: ‘This is how I see it’ or, ‘This is my interpretation of what participants were discussing’
Step 2: B to A. Co-researcher B responds to co-researcher A’s statement. For example: ‘I have another way of seeing it’ or, ‘I see your point, but my interpretation is slightly different’ or ‘focuses on a different issue’.
Step 3: Co-researcher A modifies to a lesser or greater extent his or her initial statement: ‘Now I see it differently’ or ‘Your point made me think in another way’ or ‘your view supports mine but adds to it’. Here, the point is not to change the initial statement, but rather to remain open to be influenced by the other’s point of view.

Javier introduced the model to *SecondAuthor*, who was keen to try it out. Both agreed to avoid what qualitative scholars call proceduralism, which means treating the steps like a baking recipe that researchers follow faithfully to ensure a successful product. They also agreed to immerse in the interviews and took notes independently prior to using the model. To support note taking, they engaged in the following analytical tasks.

First, they registered what Mitchell and Clark (2021) called ‘data earworms’. This concept refers to the repetition of participants’ quotes in one’s thinking, like when a line of a given song gets stuck in one’s head. Data earworms can be a single catch phrase from a participant, or a variation of the same phrase uttered by different participants, for example. The second task entailed questioning data. For example, one key question was: What does the interview data say to the previous Delphi data? The third task consisted of identifying key messages that could inform the programme design straightforwardly. The last analytical task involved recognising instances in which one expert’s voice could be heard in the voice of another expert. In dialogical inquiry, this phenomenon is called resonance (Frank, 2011). Through resonance, Frye (1982) noted, ‘a particular statement in a particular context acquires a universal significance’. Resonance was explored in relation to each dyad, between different dyads, and between dyads and other voices outside the interviews (e.g. as part of the academic literature and in policymaking contexts). Following from this process, the co-researchers met via Zoom to share and discuss their findings and interpretations. In such discussions, they actively tried to avoid the consensus fallacy, which refers to the idea that an interpretation is valid when it can be followed by all the researchers. As Smith and McGannon (2018) argued, the chances of agreement among researchers rise when interpretations are superficial and thin. This does not mean that agreement should be avoided at all costs. Doing so might be as misleading as forcing agreement. As such, the point was to dialogue around both agreement and disagreement, in order to challenge each other’s interpretations and avoid settling for the lowest common denominator. The three-step dialogical model highlighted earlier proved to be a useful tool in this respect.

The co-researchers recorded and studied the analysis sessions that took place over Zoom and opened a document that both could access to comment and respond to each other. That is how, together, slowly, they wrote and re-wrote the results, until a complete draft was produced. This draft was discussed with members of the co-production group. Shaesta and Brett were too part of this feedback. Finally, member reflections were used (Smith & McGannon, 2018). In particular, four participants gave us their feedback, which helped us adjusting concrete parts of the manuscript.

## Results

The data afforded by the interviews helped us enhance the knowledge base built through the Delphi study in relevant ways. Below, we present a selection of empirical findings that illustrate how.

### Contents to Include in the Education and Training Programme Prototype

In the Delphi study, experts did not reach consensus to include the social model of disability in the programme. This was heavily problematised in the interviews. The social model is a framework that conceptualises disability as ‘the disadvantage or restriction of activity caused by a social organisation that does not take into account people who have impairments and excludes them from community life’ (Haegele & Hodge, 2016, p. 197). During the analysis of interview data, ‘the social model needs to be there’ was a data earworm for us. The importance of not only including but privileging this content is illustrated in the following choral quote which include textual cites from all the interviews:

I’m liking all the ticks (☑), but there’s some really huge things here. So, the social model and health inequalities is the one that really gets me. This should absolutely be over the consensus threshold. I can’t believe it's not there. I think it’s really hard to understand things like barriers if you dion’t actually understand social model. If there is ignorance about health inequalities for people with learning disabilities, then there’s even more reason it should be there. Social model up front. It underpins everything.

In a similar fashion, some experts found personal budgets way more important than highlighted in the Delphi. For example, Ben said: ‘if you look at the care act, physical activity is linked to the assessment, and the assessments are linked to personal budgets, so it seems difficult to understand how it doesn’t get consensus’. Following discussion about this result, several experts suggested that both the social model and personal budgets might not have reached consensus due to the Delphi participants’ assumption that SWs would already know about these contents. In this sense, Tim suggested that a ‘checking should be done beforehand to make sure that the assumed knowledge is there’. Sean contended: even if students know about this, it is important to teach about how the social model is embedded in practice and how personal budgets can be use specifically to help disabled do PA.

Looking at Figure 1, the absence of a tick (☑) next to ‘being active at home’ also called experts’ attention. They highlighted four reasons why this content should be incorporated in the programme. The first is contingent: currently, the Covid19 pandemic raises concerns over the safety of being active outdoors. As Naivi said: ‘People are still anxious about going out’. The second refers to the environmental barriers that disabled people face: because there are so many barriers including lack of transport and inaccessible gyms, it is more practical for some to stay home. The third is that doing exercise at home is safe and cost-effective. The fourth is that many disabled would benefit to be active at home before going outside. Eva used her lived experience of disability as a case in point; doing PA at home prepared her to take pleasure in activity outdoors. The collection of at home workouts provided by Get Yourself Active on their website was highlighted as a useful resource for signposting. Other resources to include in the training resources were recommended in the interviews, such as the content from Richmond group of charities, We are Undefeatable and the Social Care Pack.

### Learning Methods and Considerations for Teaching the Training Programme Contents

The Delphi study positioned interactive discussions as the most important method to deliver training the programme. This was echoed in the interviews. However, what was considered here was the question of who should be involved in the cited interactive discussions. The experts argued that people with lived experience and not just students should be involved. ‘That’s going to be the most powerful tool in teaching SWs’, asserted Bob. His dyad agreed: ‘I would hope that things like What PA means to disabled people is delivered and led by people with lived experience’; and added: ‘Non-disabled people talking to non-disabled people doesn’t challenge assumptions’. In line with these reflections, the item ‘invited talks and blogs’ was reassessed as much more important as in the Delphi results. For example, Fiona commented that invited talks and blogs can be helpful to gather a variety of voices and ‘opening up the interactive discussions’.

Another method that can help open up discussions and ‘get examples of how people deal with things’ (Tim) is case studies. Like in the Delphi, this method was regarded indispensable, but three messages were added to knowledge when interviews were conducted. First, case studies should be presented in a way that is digestible for SWs, as they can find a booklet full of detailed case studies overwhelming (Bob). Second, case studies should not merely present cases of success, but show as well how things can go wrong (Sean). Finally, storytelling should be used as it is an effective way of presenting case studies and ‘bring them to life, so that they have an emotional impact on SWs’ (Ceri). Bob shared an example. First, students would be presented with an example of the physical activity trap, such as this video: https://www.youtube.com/watch?v=JWpTxvtg744. Then they would be asked: What can a social worker do to change this? Bob recognised that this kind of question is difficult to answer, and yet unavoidable. Asking uncomfortable questions throughout the programme, stated Sue, is imperative.

Finally, the experts drew their attention to the item named ‘scenario-based learning’. This item was defined in the Delphi in terms of giving SWs the chance to visit the scenarios where disabled people do PA and observe how they do it. All experts gave positive arguments for why this method reached consensus, except one, Sue. She raised an important caveat:

If the social worker wants to come and observe me having my gym session, I wouldn’t be fine with that (…) They could learn by asking me (…) Disabled people are constantly observed by other people. If physical activity is for fun… it’s another space where someone is going to come and look at you. I’d be like: no, thank you.

We mentioned this caveat in the interviews that followed the one involving Sue. This includes the interview involving Eva. Before hearing about Sue’s words, Eva sustained that scenario-based learning could have a ‘massive positive impact’. After hearing the caveat, Eva added that that gaining consent would indeed be essential if this learning method is to be employed. The above resembles the three steps in the dialogical model that Javier and Chris used throughout the analysis. Eva said something, Sue’s response was introduced, and then Eva added to her initial statement to recognise Sue’s caveat.

### Barriers that Can Compromise the Success of the Teaching Programme (and How to Address Them)

In the Delphi study, experts agreed that diverse barriers needed to be considered, but they did not have the chance of discussing how these barriers can impact the programme, and how they might be overcome. The interview gave them this opportunity. First, the experts pointed out that many of the barriers that achieved consensus in the Delphi could be addressed *before* the training programme content is introduced. Their propositions can be summarised in two related tactics. The first is presenting a strong rationale for why the training content matters for people taking the course. That would help tackling barriers such as lack of understanding and, by association, lack of interest and commitment. As Bob argued, ‘If social work students understand [the programme rationale] better, they would be more interested’.

The second tactic is about appeasing SWs. Before learning how to promote PA, SWs must feel assured that the programme will not be asking them to shift their focus from wellbeing, but the opposite. They need to *appreciate* that PA is a means to take care of people’s social, mental and physical wellbeing. Then, SWs need to be made aware that they are not alone. They are not being asked to get disabled people active on their own and with the lack of resources they often limit them. They are seen and should see themselves as a part of a wider gear which includes other messengers, including occupational therapists and physiotherapists. Experts suggested that mentioning the word ‘multiagency’ would be useful to communicate this point, as SWs are familar with it. Equally significant is to avoid coercing SWs into PA promotion and, instead, to share affirmative messages like: promoting PA ‘will actually make your working day more enjoyable, more productive, easier. You can have exciting, fruitful conversations with people about making positive changes in their life’ (Sean). Moreover, SWs should also be convinced that the training and the future work delivering PA messages ‘will not be hard work for them’ (Rafa). They are not expected to act as physicians, coaches, and psychologists. A caveat should accompany this message: embedding PA as part of everyday conversation as a social worker will not be automatic. It will take time, practice and reflection. Therefore, the training leaders ‘need to tell SWs that they will be allowed to try and fail and try again’. All experts emphasised the importance of this point.

In addition, experts suggested that many of the barriers privileged in the Delphi can be tackled *through* delivering the training content. These include lack of understanding, interest, commitment and confidence, but also the stereotyped views that SWs may have on disability (e.g. the perception that disabled person are too fragile to do PA). For Sue, challenging potential assumptions or myths about disability during the training is essential. It would help address other barriers highlighted in the Delphi, including risk aversion. The experts directly connected with PA insisted that it is safe for disabled people to do PA. To gain awareness about that, SWs need to be aware of the evidence stating that PA benefits outweigh risks for disabled people and people living with long term conditions (Reid et al., 2022). More importantly, said the experts, SWs need to listen to what disabled people say they can and cannot do. In this respect, bad communication skills can be a dangerous barrier, which in turn means that ‘communication skills is a vital content of the programme’ (Tim)

In the same way the programme needs to challenge assumptions about disability, it must do the same with PA. This can be done through core contents such as ‘Definition and types of PA’. When delivering this content, the experts suggested, it is important to stress that PA is much more than sports and competition. It is about moving the body in everyday contexts, and it might involve ‘open the front door and do some gardening’ (Fiona), ‘dancing in the kitchen’ (Eva) or ‘doing some cultural activities with others’ (Sean and Ben). Overall, experts recommended telling SWs that PA can be to feel hostile to people, but also kind to them, and that reviewing assumptions of what is PA can help them find the kindness in it. This is especially so when considering that some SWs might have had negative PA experiences in the past, which fed into them and removed them from all contexts of PA.

Finally, the experts commented on the barriers for long-term success that do not lay on the feet of SWs alone. They maintained that, even though it is key that SWs and HCPs work together, both workforces often have an uncooperative attitude. Bob commented that HCPs and SWs ‘do not have honest discussions because everyone feels that they are going to be told: you’re wrong’. Likewise, Naivi affirmed that SWs ‘do struggle working with HCPs. Not all the time we are in the same boat’. Optimistically, Eva suggested that PA can help address this tension and ‘be the space in which HCPs and social care professionals get together’. For this to be possible, Ben argued that curriculums should converge: ‘the more joint teaching we can do, the better’. That curriculums are different might be problematic ‘because we know the hierarchy of professions, and social care is not at the top’. (Sue). In view of these points, addressing power imbalances between SWs and HCPs appears to be the first necessary step. In this respect, it would be important to ‘identify the things they have in common. Identify shared goals and aspirations. Then there’s room to accept the differences there. You don’t flag them up. You don’t highlight the differences before you highlight the commonalities’. (Sean). To conclude this discussion, dyads highlighted the importance of researching how HCPs and SWs work together now, and how education could help them work better in the future. ‘It’s something we will have to think about’ is another data earworm that has stayed with us.

Secondly, experts suggested that employers, senior managers and national organisations such as Social Work England need to endorse and back the training programme to ensure its progress. As Ben said, ‘If it does not come from the top, then it’s not going to be used on the ground level’. In practice, this means recognising the work and giving SWs’ incentives to promote PA, but also ‘being more flexible to give SWs time to try out new things' (Rafa).

## Concluding Discussion

The curiosity-driven qualitative research presented in this paper followed a previous Delphi study. As highlighted, the Delphi study was conducted in order to design the contours of a training programme prototype. This paper has provided us with additional layers of knowledge on experts’ opinions that could not be obtained through the Delphi method. Moreover, it has allowed us to rectify seemingly clear expert agreements on what the MSW training programme prototype should include, and what needs to be done to achieve long-term impact. We have used the new qualitative evidence to refine the initial iteration of the programme prototype. Sections have been added to our programme summary, and the structure of our teaching resources have evolved. Overall, a much more nuanced output has been developed that was considered more relevant, useful and useable.

Importantly, though, this research has not resulted in a final output. On the one hand, a dialogical philosophy does not tolerate finalising claims. A finalising claim says the last word about what something is or can become, preventing it from changing and evolving over time (Frank, 2005). By contrast, dialogical inquiry ‘aims at increasing people’s possibilities for hearing themselves and others. It seeks to expand people’s sense of responsibility (a Bakhtinian pun on response) in how they might respond to what is heard’ (Frank, 2012, p. 37). In this sense, the results of this research are not meant to establish a definitive design for the training programme. That is why we have been calling it training programme prototype. The design of the training programme that we have created drawing on the literature, the Delphi study, and now this interview study, remains open to more voices. This includes the voices of people with lived experience, like those who experience disability. To witness such voices, knowledge cafés have been recently conducted. The knowledge café, or what is also called World Café, is a research activity that allows having unstructured conversations with and learn from marginalised voices (Netherway, et al., forthcoming). The 86 people with lived experience who have participated in the MSW knowledge cafés engaged with the prototype iteration derived from this research, sometimes reinforcing its components, sometimes challenging them. After the cafés, some elements have stayed and are part of the new iteration. Others have been amended or expanded, and a few have been removed. The latest iteration of the programme prototype is now being used to teach social work undergraduate students and SWs in continual professional development training. Staying with dialogical inquiry, observations of the teaching and interview-based conversations with students and lecturers about their experiences of participating in the programme testing will inform the succeeding prototype iteration.

Besides generating empirical insights to advance the MSW project, this article has provided contributions that may be of interest to different audiences. These audiences include researchers and practitioners interested in co-production, and health and social care education. For instance, this research has illustrated how co-produced, dyadic and data-prompted interviews can be conducted, and how a co-produced analytical process might look like in action. Furthermore, it has presented original knowledge on what kind of considerations revolve around PA promotion training for SWs, and in doing so, it has helped us understand the contemporary condition of both PA promotion and social work. The process may also be useful for others to help know how to create an evidence-based training programme for other professionals, such as occupational therapists, nurses and physiotherapists. However, the most significant contributions of this article are discussed below.

In the first place, this research has showed that dialogical inquiry and co-production are a good fit. This is (at least) because of three interrelated reasons. Firstly, constant dialogue is a necessary condition for the ethical and practical success of co-production. Secondly, dialogue, or at least how dialogue is idealised in dialogical inquiry (e.g. Frank, 2005), refuses hierarchies between people as well as the ‘tyranny of the last word’ (Levinas, 1998, p. 141) – which is conventionally uttered by the researcher. Thirdly, dialogical inquiry offers ways of doing analysis that are reasonably accessible for non-academics. Certainly, the ideas and language of dialogical analysis are sophisticated, and can become as hard to reach as any other complex form of qualitative analysis. We could even call dialogism ‘high theory’. But, as Strom (2018) argued, some exclusionary iterations of high theory can and should be interrupted. Indeed, some core elements of dialogical analysis such as the identification of resonances can be made accessible without losing all their substance. In the future, it would be worth formalising the dialogical foundations of co-production. Although excellent scholarship is being done to theorise co-production which mentions the idea of dialogue, this task is yet to be done.

In the second place, the research has called on us to question whether a Delphi study alone can be the basis of any educational programme, curriculum or policy agenda. Although there are good reasons why consensus-building methods such as the Delphi are privileged in policy research, the present study has revealed that the pursuit of consensus is likely to invest a superficial agreement with righteousness, brush minority views under the carpet, fail to collect concrete recommendations, and miss the heuristic potential of conflict and relational thinking. In light of that, using qualitative methods after a Delphi study can be very important – not only to recognise dissent (Shrier, 2021) but also to tackle it properly. Yet it is unlikely that the anecdotal use of qualitative techniques allows exploring conflict in depth. For example, from a qualitative stance it is inadequate to conduct short interviews and a content analysis, whereby data are coded and analysed numerically. Although this analysis might be a good addition within Delphi studies (e.g. Krijtenburg-Lewerissa et al., 2019), it is unlikely to capture the complexity and nuance of expert knowledge. Against this, it is critical to engage with what Braun and Clarke (2013) call ‘Big Q qualitative research’. This refers to research that applies qualitative methods within a qualitative paradigm, rather than a positivist one. Big Q qualitative research thus avoids converting qualitative data through a quantitative framework, proceduralism and taking things at face value. It asks questions through a qualitative lens, including about why things may be that way and how they could be otherwise. Vindications for Big Q qualitative research and co-production pile up. Here, we have added a modest example that can be used to support such vindications. We hope it is useful and used.
